# The Role of Jasmonates in Floral Nectar Secretion

**DOI:** 10.1371/journal.pone.0009265

**Published:** 2010-02-19

**Authors:** Venkatesan Radhika, Christian Kost, Wilhelm Boland, Martin Heil

**Affiliations:** 1 Department of Bioorganic Chemistry, Max Planck Institute for Chemical Ecology, Jena, Germany; 2 Departamento de Ingeniería Genética, Centro de Investigación y de Estudios Avanzados (CINVESTAV) Irapuato, Guanajuato, México; Iwate University, Japan

## Abstract

Plants produce nectar in their flowers as a reward for their pollinators and most of our crops depend on insect pollination, but little is known on the physiological control of nectar secretion. Jasmonates are well-known for their effects on senescence, the development and opening of flowers and on plant defences such as extrafloral nectar. Their role in floral nectar secretion has, however, not been explored so far. We investigated whether jasmonates have an influence on floral nectar secretion in oil-seed rape, *Brassica napus*. The floral tissues of this plant produced jasmonic acid (JA) endogenously, and JA concentrations peaked shortly before nectar secretion was highest. Exogenous application of JA to flowers induced nectar secretion, which was suppressed by treatment with phenidone, an inhibitor of JA synthesis. This effect could be reversed by additional application of JA. Jasmonoyl-isoleucine and its structural mimic coronalon also increased nectar secretion. Herbivory or addition of JA to the leaves did not have an effect on floral nectar secretion, demonstrating a functional separation of systemic defence signalling from reproductive nectar secretion. Jasmonates, which have been intensively studied in the context of herbivore defences and flower development, have a profound effect on floral nectar secretion and, thus, pollination efficiency in *B. napus*. Our results link floral nectar secretion to jasmonate signalling and thereby integrate the floral nectar secretion into the complex network of oxylipid-mediated developmental processes of plants.

## Introduction

Nectar is an aqueous plant secretion that mainly contains sugars and amino acids [1], [2]. Many higher plants produce nectar in their flowers to attract insects or vertebrate pollinators, which transport pollen from one plant to another, thereby enabling outcrossing. Outcrossing contributes to the evolutionary success of angiosperms and lack of pollination often limits fruit yield [3]. Nectar rewards immensely influence pollinator behaviours such as visit frequency, number of flowers probed, probe time per flower, and also the movement of the pollinator after leaving the plant [4]. Flowers secreting more nectar are more successfully pollinated and higher levels of nectar may be one key to enhanced outcrossing in response to insect visitation [5]. Hence, floral nectar is involved in a highly important interaction among plants and animals. Despite these central ecological, evolutionary and economic functions, little is known on how plants control nectar secretion physiologically [6].

Variability in nectar secretion by environmental and physiological factors [7] and the dynamic regulation of nectar volume by reabsorption [8] and refilling of nectaries upon removal [9] have been reported [3]. Most recently, an extracellular invertase has been identified as a factor that is causally involved in nectar secretion in *Arabidopsis thaliana* flowers [10]. However, little is known about the hormonal regulation of floral nectar.

Here, we investigated whether jasmonates are involved in the control of flower nectar secretion. Jasmonates (term collectively used for all bioactive representatives of the jasmonate family) control central processes in plants such as root growth, defence, tendril coiling and reproduction [11], [12]. In flowers, jasmonic acid (JA) plays multiple roles that are related to general developmental processes [13], [14]. On the one hand, negative effects of jasmonate on flower opening and bud initiation have been reported for *Pharbitis nil* and *Nicotiana tabacum* [13], [15]. On the other hand, JA appears to be necessary for pollen development and anther dehiscence in *Arabidopsis* [16]. Moreover, a tissue-specific synthesis of JA in flowers has been described [17]–[20]. Much less is known on the role of JA for nectar secretion. JA, its precursors and its derivatives orchestrate plant defence responses [12], including the secretion of extrafloral nectar [21], [22], but their putative role in the regulation of floral nectar secretion has apparently never been considered.

**Figure 1:**
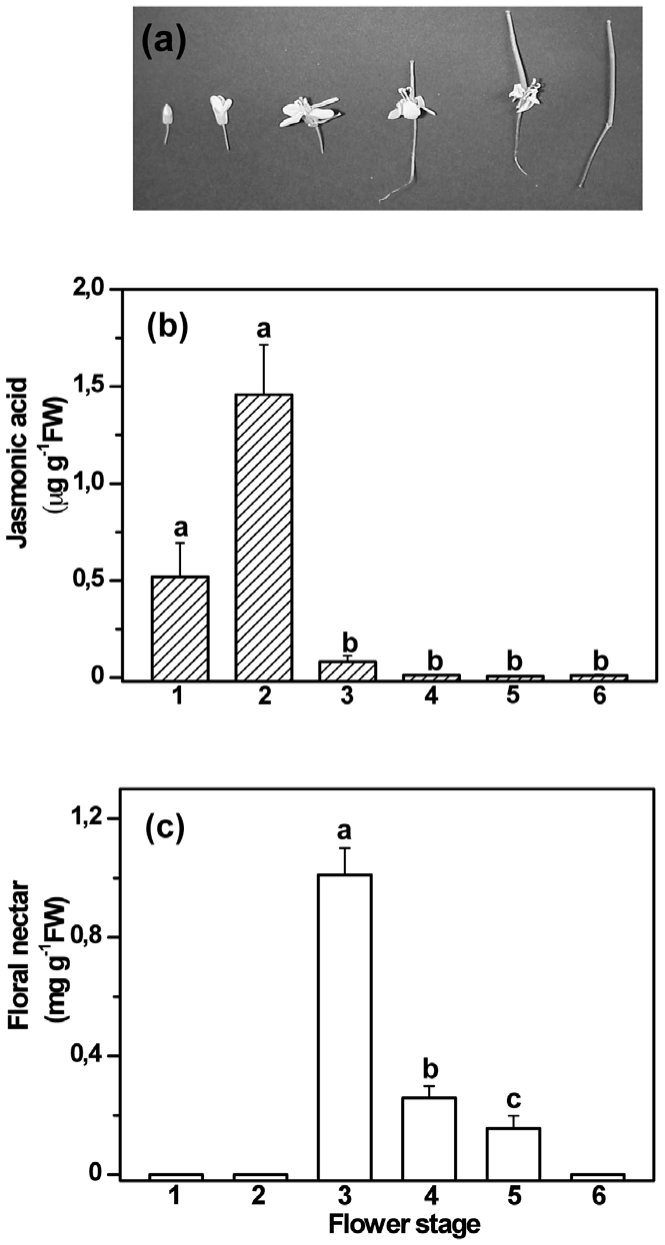
Ontogenetic changes of nectar secretion and endogenous JA in flower tissue. Panel A: Flower stages 1–6 as defined for the present study. Panel B: JA concentration (mean ± SE) is displayed in ng JA per g fresh mass. Different letters indicate significant differences among different stages (LSD post-hoc test after univariate ANOVA, P<0.02, n = 5). Panel C: Nectar secretion (mean ± SE) is given in mg soluble solids per g fresh mass of the flowers. Different letters indicate significant differences among stages (LSD post-hoc test after univariate ANOVA, P<0.01, n = 10). Only the flower stages with nectar secretion (3–5) were included in the post-hoc test in order to avoid inhomogeneity of variances due to zero-production in stages 1, 2 and 6.

To investigate whether floral nectar secretion is regulated via jasmonates, we used *Brassica napus* (canola or rapeseed) as experimental system. In this species, the nectar secretion is highest in fully-open flowers (Figure 1). *B. napus* is an important agricultural crop that attracts insect pollinators [23]. Nectar secretion has been shown to have positive effects on fruit ripening and seed germination rate, and it reduces the flowering period [24]. First, we investigated the relationship between ontogenetic changes in nectar secretion and endogenous JA levels. Assuming that the secretion of floral nectar secretion is affected by JA during flower development, we hypothesised that the temporal secretion pattern should correlate with the endogenous concentrations of JA in the flower tissue. We also predicted that any temporal changes in the JA content of the flowers should precede floral nectar secretion. Second, we exogenously applied to the flowers JA, the JA-amino acid conjugate jasmonoyl-isoleucine (JA-Ile), its mimic coronalon and phenidone (an inhibitor of endogenous JA synthesis). We predicted that application of JA or its mimics should induce EFN secretion, whereas phenidone should have an inhibitory effect. Finally, we investigated whether systemic, JA-dependent responses to leaf damage interfere with floral nectar secretion. Jasmonates are known to be systemically transported [21], [25], [26] and their application to – or induction in – leaves might therefore also affect floral nectar secretion. The results of our study represent a first step towards understanding the hormonal control of nectar secretion in flowers and its putative interference with other plant functions.

## Results

### Ontogenetic Changes in Nectar and Endogenous JA Levels

The developmental floral stages as defined for this study are presented in Fig 1. We classified the flowers morphologically into six stages starting from the very young bud (Stage 1) to the withered flower (Stage 6) as described in refs [27], [37]. We distinguished the following six stages of flowers: stage 1 - loose bud, petals not expanded, stage 2 - corolla opening, beginning of anthers dehiscence, stage 3 - corolla fully expanded, full pollen exposure; stage 4 - corolla completely open after pollen exposure, stage 5 - shrivelled corolla, no pollen and stage 6 - withered corolla. Each flower remains open for about 3–4 days. Nectar secretion starts when the corolla is open in stage 2 and increases in the next stage when the corolla is fully expanded and the pollen is exposed and continues till stage 6 [37]. In our experiments, maximum amounts of nectar were produced when flowers were fully opened (stage 3, see Fig. 1, LSD post-hoc test after univariate ANOVA, P<0.01, n = 10). Endogenous JA levels showed a peak shortly before nectar secretion was highest (stage 2, see Fig. 1, LSD post-hoc test after univariate ANOVA, P<0.02, n = 5). The levels of endogenous OPDA (12-oxo-phytodienoic acid), the precursor of JA, were found to be approximately 25–50 ng per g fresh weight in stages 2, 3 and 4 and in the other stages of flower development the level of OPDA was lower than 20 ng.

### Induction of Nectar by JA

**Figure 2:**
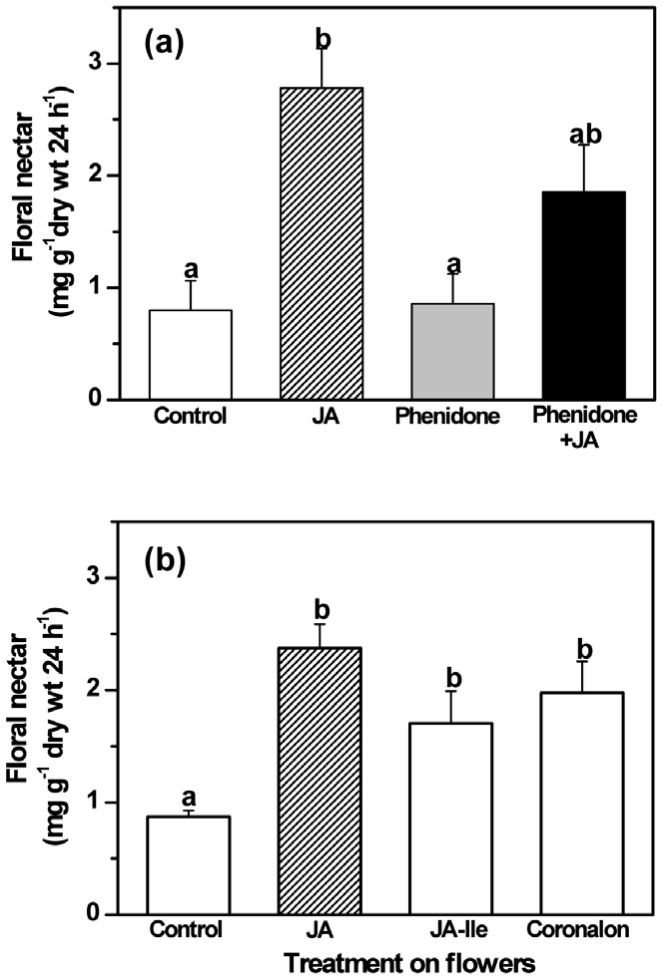
Changes in floral nectar secretion rate in response to different treatments. Panel A shows the consequences of an inhibition of *de novo* biosynthesis of JA. Different treatments (expected response in brackets) were: untreated (control levels), JA (increase), phenidone (reduced) and Phenidone + JA (restored). Nectar secretion rate (mean ± SE) is given as mg soluble solids per g dry mass of the flowers per 24 h. Panel B: Induction of nectar secretion with JA, JA-Ile and coronalon. Nectar secretion rate (mean ± SE) is given as mg soluble solids per g dry mass of the flowers per 24 h. Different letters indicate significant differences among treatments (LSD post-hoc test after univariate ANOVA, P<0.01, n = 7 and 8, respectively).

Exogenous application of 1mM JA significantly increased nectar secretion after 24 h in comparison to control plants, which had been sprayed with water (Fig. 2a, LSD post-hoc test after univariate ANOVA, P<0.01, n = 7). Glucose and fructose were the major constituents of the nectar and the G∶F ratio was in the range of 1.2–1.3 (Table 1). The sucrose concentrations were very low or undetectable. The nectar, thus, represents an hexose-dominated nectar according to the classification proposed by Baker & Baker [35]. No changes in nectar sugar composition were observed after JA treatment (Table 1). The effect of JA induction thus appears to be quantitative rather than qualitative. Next, we treated the flowers with phenidone, an inhibitor of lipoxygenases [38] that blocks endogenous JA synthesis. Phenidone treatment reduced nectar secretion to control levels after 24 h (Fig. 2a, LSD post-hoc after univariate ANOVA, P<0.01, n = 7), but high secretion rates could be restored by additional exogenous application of 1 mM JA following the phenidone treatment (Fig 2a). Application of phenidone did not lead to lower nectar levels than seen in control plants; hence attempts were made to treat plants with phenidone at early flowering stages (stage 1 or 2). However, this treatment led to delayed flower opening and not to a further decrease in nectar levels. Additionally, no significant reduction in the floral nectar secretion below control levels was observed when higher concentrations of phenidone (6 or 10 mM) were used.

**Table 1:** Sugar composition of floral nectar after different treatments.

Treatment	Sugars (%)		G-F ratio
*of leaves*	Glucose	Fructose	
Tap water	56.6±5.8	43.3±4.8	1.3
JA	47.9±1.5	52.1±11.2	0.92
Mechanical damage	57.3±5.6	42.7±4.3	1.34
Specialist herbivore (*P.rapae*)	50.3±2.8	49.7±5.4	1.01
Generalist herbivore (*S. littoralis*)	56.7±5.6	43.2±5.5	1.31
***of flowers***			
Tap water	54.7±2.2	45.3±2.0	1.21
JA	55.9±3.7	44.1±3.0	1.27

Relative sugar concentration (mean ± SE) is given for 10 plant replicates. Nectar from 4–5 flowers per plant were pooled in all cases.

### JA Conjugates Induce Nectar Secretion

JA is transformed into a variety of metabolites such as methyl JA, hydroxyl JA and amino acid conjugates after its biosynthesis [12]. Recent reports on the jasmonate (ZIM) domain (JAZ) family of transcriptional repressors of jasmonate signaling have established that jasmonoyl isoleucine (JA-Ile) is a crucial regulatory signal for JA related responses [39]–[41]. In order to investigate whether floral nectar secretion responds to known central regulatory factors of the octadecanoid signalling pathway, we treated the flower tissue with JA-Ile and its structural mimic coronalon [30], [32]. Treatment with both JA-Ile and coronalon led to a significant increase in nectar secretion as compared to control plants (Fig 2b, LSD post hoc test after univariate ANOVA, P<0.01, n = 8). There was no significant difference in the nectar production among the treatments with JA, JA-Ile and coronalon.

### Signalling Conflicts between Anti-Herbivore Defence and Floral Nectar Secretion

**Figure 3:**
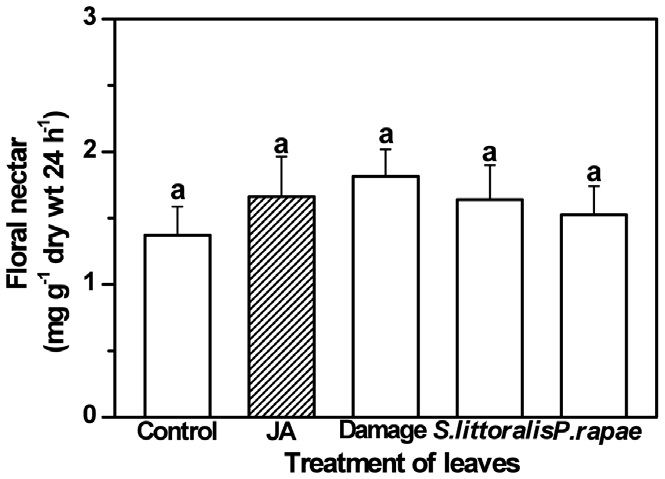
Nectar secretion rate in response to natural and mimicked leaf herbivory. Herbivory of leaves was mimicked by the exogenous application of JA, mechanical damage, or inflicted by either generalist (*Spodoptera littoralis*) or specialist (*Pieris rapae*) herbivores. Nectar secretion rate (mean ± SE) is given as mg soluble solids per g dry mass of the flowers per 24 h. No significant differences among treatments could be detected (LSD post-hoc test after univariate ANOVA, P>0.05 for all comparisons, n = 10).

To study whether systemic defence signalling interferes with the observed JA-mediated induction of floral nectar, we treated the leaves of *B. napus* with JA, mechanical damage and natural herbivores, treatments which are all known to increase endogenous JA levels [11], [12], [26]. No detectable effect on floral nectar secretion was observed when leaves of *B. napus* were subjected to application of JA, mechanical damage and leaf damage by generalist (*S. littoralis*) and specialist (*P. rapae*) herbivores (Fig. 3, LSD post-hoc test after univariate ANOVA, P>0.05, n = 10). Even maximal herbivore damage afflicted by at least 2 larvae per every leaf did not affect nectar secretion in flowers. The nectar's sugar composition remained unchanged after all of these treatments (Table 1). Nectar was predominantly hexose-rich and the glucose∶fructose ratio was 0.9–1.3, similar to the nectar composition that had been observed in the other experiments.

## Discussion

As a first step to investigate whether the phytohormone jasmonic acid (JA) is involved in the secretion of floral nectar, we followed endogenous JA levels and the amounts of nectar secreted during flower ontogeny in *Brassica napus* plants. A burst of endogenous JA preceded the maximal nectar secretion, suggesting that JA controls nectar secretion in flowers in the same way as it induces the secretion of defensive extrafloral nectar [22]. The observation that exogenous application of JA to the flowers of *B. napus* significantly increased the production rate of floral nectar corroborated this interpretation. When endogenous JA synthesis was inhibited at the stage of highest nectar secretion by application of phenidone, nectar secretion decreased to control levels. Phenidone only inhibits one early enzymatic step in the octadecanoid cascade [38] and thus reduces the *de novo* synthesis of endogenous JA, but it does not affect JA-concentrations that are already present in the tissue [22]. Our results indicate, therefore, that basal JA levels were sufficient to allow a background nectar production. Even higher concentrations of phenidone (up to 10 mM) did not significantly reduce nectar secretion further and high nectar secretion could be restored when JA was applied in addition to phenidone (Fig 2a). Both observations exclude a direct inhibitory effect of phenidone on nectar secretion and support a positive effect of JA or its derivatives on nectar secretion rates in *Brassica napus* flowers.

The endogenous JA level peaked in the flower stage 2 (Fig. 1), which precedes the stage with the highest nectar secretion (stage 3). Because JA is subject to natural turnover rates, blocking the *de novo* synthesis of JA using phenidone at earlier stages of flower development (stages 1 and 2) likely would have reduced the JA levels in the following stages even below the levels that ocurred in control plants. Unfortunately, applying phenidone to earlier stages of flowering such as stage 1 or 2 delayed or even ceased flower opening and was, thus, not feasible in the context of the present study. Jasmonic acid is a multifunctional growth regulator in plants that modulates many developmental processes [12] and has repeatedly been reported in the context of flower development. In *Arabidopsis thaliana*, flower development is linked to JA biosynthesis [34] as shown, for example by *coi1* mutants, which are defective in JA-signalling and male sterile [18]. The triple mutant *fad3fad7fad8* has also been shown to have an anther-dehiscence defective phenotype: this mutant lacks the fatty acid desaturase, which catalyses the removal of two hydrogen atoms from linolenic acid to generate the free linolenic acid, an important precursor for JA biosynthesis [19]. Recently Sanders *et al.* have reported a similar result in the mutant of DELAYED DEHISCENCE 1, that encodes an enzyme, 12-oxophytodienoate reductase, which catalyzes the formation of the JA-precursor OPDA [20]. Unfortunately, none of these studies reported nectar secretion rates, likely due to the small size of *Arabidopsis* flowers. Furthermore, far-red light inhibited flower opening in *Pharbitis nil* [13] and the same wavelength can inhibit the sensitivity of JA-regulated genes to jasmonates and thus, suppress their expression even when JA is present [42], [43]. In a recent study on *Brassica napus*, exogenous application of MeJA at early stages of flower development affected flowering time, flower morphology and the number of open flowers [44]. Similarly, exogenous MeJA interfered with normal flower development in *Chenopodium rubrum* [45]. In our study, we found (i) that increased JA levels preceded the highest nectar secretion rate, (ii) that inhibiting endogenous JA synthesis at early stages of flower development negatively interfered with flower development and (iii) that exogenous JA at the stage of highest natural nectar secretion further increased secretion rates. All these observations are in line with our interpretation that JA at earlier flowering stages is essential for normal flower development and at later stages involved in the control of nectar secretion.

Are the increases in nectar secretion seen after elicitor treatment in our study within a natural range? Quantitative dose-response relationships were found in the induction of extrafloral nectar production in *Macaranga tanarius* plants that were sprayed with JA [22]. In our study, the concentration of elicitors was 1mM in all cases and the same concentration elicited responses within natural ranges when used to induce other species, whereas higher concentrations are known to have phytotoxic effects [46]–[49]. We, thus, conclude that the maximum rates of nectar secretion, which we observed in JA-treated flowers, were still within ranges that may also occur in nature.

Research on jasmonate signalling recently experienced a significant breakthrough with the discovery of a family of JAZ (jasmonate ZIM-domain) proteins [39], [40]. Jasmonic acid does not directly induce gene activity, rather, the JA-amino acid conjugate jasmonoyl–isoleucine (JA-Ile, see ref [50] binds to the COI1 (coronatin-insensitive 1)-unit of an E3 ubiquitin ligase complex termed SCF^COI1^ (for Skip/Cullin/Fbox – COI1), which targets JAZ-proteins for ubiquitination and thus their rapid degradation [39]. When we treated the flowers with JA-Ile and its structural mimic coronalon, an increased nectar flow was observed. These results demonstrate that the signalling cascades, which control floral nectar secretion, are very similar to those involved in jasmonate-responsive gene expression in tomato and *Arabidopsis* [41], [50].

Plants do not only interact with pollinators, but also with other insects, many of which are detrimental to the plant since they feed on plant tissue. One of the remarkable features of plant defences against these herbivores is that they are often inducible, with JA acting as the central signalling molecule. Considerable evidence exists to support the systemic induction of defence responses in plants when only certain plant parts are attacked [51] and recent data [26] support that jasmonates can move through phloem and xylem to induce defences in distant plant parts. Such a long-distance transport of JA or other jasmonates could cause signalling conflicts between leaves and flowers. Does, therefore, damaging the leaves of *B. napus* and the resulting release of jasmonates from damaged leaves interfere with the nectar secretion in flowers? Increasing nectar secretion in flowers in response to leaf herbivory would demand more resources to flowers, which could otherwise be allocated to leaf defences. On the other hand, decreasing nectar secretion would lower the chance of pollination, which becomes even more essential in time of leaf damage or stress. Recently, Bruinsma *et al* investigated effects of JA treatment on leaves of *B. nigra* upon pollinator preferences [49]. They observed no change in pollinator preference and rates of flower visitation, but saw a decreased nectar secretion in JA treated plants. In our case, we found no difference in floral nectar secretion with different treatments on leaves. However, in their study, Bruinsma *et al.* collected nectar after 2 days of treatment, a time span that possibly was enough to reduce photosynthetic activity that thereby result in a shortage of resources required for nectar production. In our study, there was no detectable effect on the floral nectar production by damage to the leaves in a 24 h time period. As it would be expected from an evolutionary point of view, defence signalling in response to leaf herbivory does not directly interfere with the regulation of floral nectar secretion.

### Conclusions

One of the major links between pollinator behaviour and plant reproductive success or crop productivity is floral nectar, whose regulation is understudied. We demonstrate that floral nectar secretion is regulated by jasmonates, plant hormones that so far have been mainly discussed in the context of plant development and defence activation. Which physiological and genetic processes are involved in the jasmonate-responsive nectar secretion remains, however, to be elucidated. The changes that we observed were quantitative, rather than qualitative ones. The jasmonate-mediated up-regulation of nectar secretion is, thus, unlikely to impair the attractiveness of nectar to pollinators, opening interesting perspectives for crops whose pollination is nectar-limited. We also found that induction of jasmonate-dependent defence responses in leaves did not directly interfere with floral nectar secretion. The mechanisms, however, by which plants achieve this highly important functional separation remain to be elucidated. Research on jasmonate signalling in plants has recently experienced major developments, and the finding of its role in the regulation of floral nectar secretion shows that important functions of jasmonates are still being discovered.

## Materials and Methods

### Plant Material and Induction of Flowers

*Brassica napus* (cv. Dwarf essex) plants were grown in Klasmann clay substrate (Klasmann-Deilmann, Geeste, Germany) under 16 h day conditions. The plants used for the experiments were 4–5 weeks old. The flowers of the plant under study have been divided into six developmental stages based on visual observation [27] as seen in Figure 1a. Each stage lasts for about 3–4 days. Nectaries of brassicacean plants are usually present in the filament bases between sepals and stamens. In *B. napus* flowers, four nectaries develop in a circle surrounding the base of the filaments [27], [28], two of which are present at the inner side of the two short filaments and two at the outer side. The nectaries at the inner side are known as lateral nectaries and the ones on the outer side as median nectaries. The median nectaries are inactive or secrete very little nectar. In our study, we collected nectar from all the nectaries.

For all experiments with fully-opened flowers (stage 3), flowers that were open for 1d were used. An aqueous solution of 1 mM JA was sprayed on the flowers until run-off and the same amount of tap water was sprayed on control plants. The spraying was repeated after 30 min, and then the flowers were left to absorb for one hour. For phenidone (1-phenyl-3-pyrazolidinone) treatment, an aqueous solution of phenidone (2 mM, Sigma-Aldrich, Germany) was sprayed two times as described for JA. The same concentration inhibited endogenous JA synthesis without causing phytotoxicity in earlier studies [22], [29]. ‘Phenidone + JA’ treated flowers received an additional spray of 1 mM JA two times after the final phenidone application. A similar procedure was used for other induction experiments with aqueous solutions of JA-Ile (1 mM) and coronalon (100 µM) [30], [31]. JA-Ile and coronalon were synthesized according to literature procedures [30], [32].

### Rearing of Herbivores and Induction of Leaves

The generalist herbivore, *Spodoptera littoralis* Boisd. (Lepidoptera, Noctuidae) was reared at 22–24°C under 14–16 h photoperiod in plastic boxes and fed on artificial diet (500 g of ground white beans soaked overnight in 1.2 l water, 9 g vitamin C, 9 g paraben, 4 ml formalin and 75 g agar boiled in 1 l of water). The specialist herbivore, *Pieris rapae* was maintained on Brussels sprout plants (*Brassica oleracea* convar. *fruticosa* var. *gemifera* cv. Rosella) at 22°C under a 16 h photoperiod. Third-instar larvae of both herbivores were allowed to feed on all leaves of the experimental plant for 24 h by placing them in clip cages (∼4.9 g, 56 mm diameter made of transparent plastic) with at least 2 larvae per cage. ‘Damaged’ leaves were wounded by puncturing all the leaves with a pattern wheel (approximately 100 holes per leaf). Similar to the treatment on flower tissues, JA (1 mM) and tap water (control) was sprayed on all leaves. All flowers were bagged in PET foil (Toppits® ‘Bratschlauch’, Melitta, Minden, Germany) to prevent direct induction of the flowers by any airborne cue that might be released from the leaves in response to these treatments.

### Nectar Quantification

The concentration of floral nectar was measured immediately after collection using a temperature compensated refractometer (ATAGO N-10E refractometer, Leo Kübler GmbH, Karlsruhe, Germany) and the nectar volume was quantified using 5 µl micro-capillaries as described in [33]. The nectar was quantified as amount of soluble solids per g dry weight of the secreting flower material per 24 h. All experiments were conducted in a climate-controlled greenhouse. Since nectar secretion was highest in the fully opened flowers, all experiments were conducted with flowers of this stage. Application of phenidone to flowers at earlier stages led to delayed or complete cessation of flower opening, probably because JA is a ubiquitous phytohormone involved in several processes, including flower development [17]–[20], [34]. Therefore, the treatment was done to fully opened flowers only.

Nectar sugar composition was analysed by gas chromatography-mass spectrometry (GC-MS). Nectars were lyophilized and silylated using N-methyl-N(trimethylsilyl)-triflouroacetamide (MSTFA). 50µl of this reagent was added to nectar samples in 100 µl of dry pyridine and the mixture was heated to 60°C for 1 h for completion of the reaction. The silylated derivatives were analyzed by GC-MS. Sugar standards (Sigma-Aldrich, Germany) were prepared similarly and the chromatographic analysis was run twice for each sample. Samples were analyzed on a GC-Trace-MS (Thermo Finnigan) using a DB-5 column (15 m×0.25 mm×0.25 µm; AllTech, Unterhaching, Germany). The temperature program for the separation started with 40°C isothermal for 3 min followed by an increase to 120°C at a rate of 10°C min^−1^ for 2 min and then an increase by 7°C min^−1^ to 250°C. The split ratio was maintained at 1∶10 with an inlet temperature of 220°C. Both glucose and fructose concentrations were determined and their relative proportions calculated [35].

### Determination of Endogenous JA Levels

In order to compare differences in the levels of endogenous JA among various floral stages, flower tissues of approximately the same fresh weight from all 6 developmental stages (Fig. 1a) were collected and the phytohormone extracted. Endogenous concentrations of JA were quantified by GC-MS as its pentafluorobenzyl (PFB)-oxime using a Finnigan GCQ ion trap mass spectrometer (Thermoelectron, Bremen, Germany) following the procedure of Schulze *et al.* [36].

### Statistical Analysis

All experiments were analysed with linear mixed-effect models with ‘treatment’ as fixed and ‘plant individual’ as random factor. LSD post-hoc tests were performed to test for between-group differences. The following variables were transformed (transformation given in brackets) to meet the assumptions of homogenous variance: endogenous JA (log x) and nectar induction experiment by JA-Ile and coronalon (1/x). All statistical analyses were performed using SPSS 13.0 (SPSS Inc., Chicago, IL, USA).
